# A Case of Pembrolizumab-Induced Myasthenia Gravis

**DOI:** 10.7759/cureus.45455

**Published:** 2023-09-18

**Authors:** Thomas I Kosick, Krima Patel, Jacob Jasinski, Bolanle Dada

**Affiliations:** 1 Internal Medicine, St. Luke's University Health Network, Easton, USA; 2 Neurology, St. Luke's University Health Network, Easton, USA

**Keywords:** pembrolizumab induced myasthenia gravis, immunotherapy toxicity, keytruda induced myositis, seronegative myasthenia gravis, keytruda®, immune-checkpoint inhibitors, immune-related adverse event (irae), pembrolizumab, myasthenia gravis

## Abstract

With the advent of new cancer treatments, immunotherapy has emerged as an increasingly promising strategy. Undoubtedly, it has pushed oncology into a new era and is providing patients with unprecedented results. As with many treatments, however, adverse effects lead to setbacks in progress. Pembrolizumab is an immunomodulating medication that functions by binding to programmed cell death protein 1 (PD-1) receptors of T cells thereby upregulating the immune system to more effectively detect and target cancer. Myasthenia gravis (MG) is a reported side effect of this medication. Our patient is an 87-year-old male with urothelial cell bladder cancer who developed MG following the administration of pembrolizumab and was treated with plasma exchange therapy. We aim to examine the existing literature concerning treatments for MG, with a particular focus on myasthenia gravis induced by pembrolizumab. We will discuss the occurrence rates and results of such instances, along with their implications for the future of these potential therapies.

## Introduction

Myasthenia gravis is a neurologic autoimmune condition characterized by muscle weakness that improves with rest. Its pathology primarily involves autoantibodies being directed against postsynaptic acetylcholine receptors causing disruption of cholinergic transmission between nerve terminals and muscle fibers. This leads to muscle weakness and fatigue, especially in those responsible for repetitive movement and breathing. Pembrolizumab is an immunomodulating medication that functions by binding to programmed cell death protein 1 (PD-1) receptors of T cells. This suppresses tumor growth by enhancing a T cell's capability of identifying and destroying cancer cells that use the programmed death pathway to evade the immune system. 

Notably, there has been a growing body of literature reporting immune-related adverse effects (irAE), including myasthenia gravis associated with the drug's use. A lack of research into the mechanisms by which these irAE come to fruition often leads to patients being treated empirically. The Society for Immunotherapy of Cancer has recommended intravenous immunoglobulin (IVIG) and plasma exchange as the standard treatment for immune-related myasthenia gravis (irMG) [1]. However, at the time of our review, no studies had been published describing this treatment's efficacy.

We present below, the case of an 87-year-old male with a history of urothelial cell bladder cancer treated with pembrolizumab, who presented with bilateral ptosis, cervical myalgia with myositis, and new-onset atrial fibrillation with rapid ventricular response and myocarditis. Our patient had a contraindication to one of the recommended treatment guidelines and was refractory to all other treatment options. As a result, he showed very modest symptomatic improvement. In our case, we found that plasma exchange alone is not an effective means of treatment for irMG, placing emphasis on the need for further studies on the treatment of irAEs as pembrolizumab becomes more commonly used.

## Case presentation

An 87-year-old male presented to the emergency department complaining of a two-week history of worsening right-sided ptosis, new left-sided blurred vision, dysarthria, cervical myalgia, and ambulatory dysfunction secondary to fatigue. His past medical history was significant for urothelial cell bladder cancer status-post resection, remote history of brain tumor resection with residual right-sided cranial nerve VII palsy, and IgA deficiency. Pembrolizumab was initiated for treatment of his bladder cancer approximately four weeks earlier with his most recent infusion being one week prior to presentation. Initial laboratory studies were significant for elevated troponins, creatine kinase, and transaminases (Table 1). EKG was unremarkable and echocardiogram did not reveal a reduction in ejection fraction or wall motion abnormalities. Imaging of the head was obtained but did not show any evidence of stroke. Abnormal laboratory studies were attributed to the recent initiation of pembrolizumab (Naranjo score of +6). Its avoidance was recommended, and the patient was discharged with plans to follow up with cardiology-oncology in one week. At that follow-up appointment, the patient reported worsening weakness and balance. Repeat laboratory studies revealed persistently elevated troponins, liver function tests (LFTs), and Creatine Phosphokinase (CPK) (Table 1). He was also noted to have a borderline tachyarrhythmia. The patient declined cardiac MRI for further workup of possible myocarditis. With the constellation of his cardiac and neurological symptoms, the patient was advised to return to the hospital for further evaluation and management. On return to the hospital, EKG showed new-onset atrial fibrillation with rapid ventricular response. Another echocardiogram was obtained but was unchanged from his prior study. The results of his initial and subsequent laboratory studies are listed in the table below (Table 1).

**Table 1:** Comprehensive Laboratory Values Values shown represent a trend in laboratory studies obtained over the first ten days ranging from initial admission through outpatient follow-up and readmission. BUN: Blood urea nitrogen, AST: aspartate aminotransferase, ALT: alanine aminotransferase, INR: international normalized ratio, GGT: Gamma-glutamyl transpeptidase, ESR: Erythrocyte Sedimentation Rate, CRP: C-reactive protein, Total CK: total creatine kinase, CK-MB: creatine kinase-myocardial band, Hct: hematocrit,  HS Troponin: High Sensitivity Troponin, TSH: thyroid stimulating hormone, T4: thyroxine, RSV: respiratory syncytial virus.

Study Name	Reference Values	December 12	December 13	December 14	December 20	December 21
Sodium	135-147 mmol/L	137	141	140	140	140
Potassium	3.5-5.3 mmol/L	3.9	3.8	3.7	4.5	4.1
Chloride	96-108 mmol/L	102	105	104	106	107
Bicarbonate	21-32 mmol/L	28	28	26	29	27
BUN	5-25 mg/dL	22	17	20	29	24
Creatinine	0.6-1.3 mg/dL	0.8	0.67	0.86	0.9	0.71
Glucose	65-140 mg/dL	194	147	166	117	117
AST	13-39 U/L	97	100	127	160	130
ALT	7-52 U/L	102	109	141	190	164
Protime-INR	0.84-1.19				1.05	
Alkaline Phosphatase	34-104 U/L	47	46	52	54	45
Total Bilirubin	0.2-1.0 mg/dL	0.67	0.69	0.76	0.77	0.64
GGT	5-85 U/L				38	
Total CK	39-308 U/L	1841				
CK-MB	0.6-6.3 ng/mL	85.4				
WBC	4.31-10.16 thousand/uL	5.94	4.9		7.03	
Hemoglobin	12.0-17.0 g/dL	11.6	11.9		12,7	
Hct	36.5%-49.3%	35	36.4		39.5	
Platelets	149-390 thousands/uL	179	196		265	
ESR	0-20 mm/hr		70			
CRP	<3.0 mg/L		3.3			
HS Troponin I 0hr	0-50 ng/L	245			235	
HS Troponin I 2hr	delta 2hr <20 ng/L	225			233	
HS Troponin I 4hr	delta 4hr <20 ng/L	218			256	
HS Troponin I Random	8-18 ng/L			294		229
TSH	0.450-4.50 ulU/mL				6.144	
T4	0.76-1.46 ng/dL				1.01	
Influenza (nasal swab)					Negative	
COVID-19 (nasal swab)					Negative	
RSV (nasal swab)					Negative	

He was started on a diltiazem infusion and subsequently transitioned to metoprolol with a good response. Neurologic exam showed restricted eye movements bilaterally with limited upgaze and bilateral ptosis (right worse than left), with decreased neck strength secondary to pain. Deltoid strength was also diminished with easy fatigability. In view of these findings, there was concern for new onset myasthenia gravis in the setting of recent initiation of pembrolizumab. MG-specific antibodies were ordered. Anti-acetylcholine (ACh)-binding, anti-ACh-blocking, anti-muscle-specific kinase (MuSK), anti-acetylcholine receptor (anti-AChR)-modulating, and anti-ENS-2 antibodies were within normal limits (Table 2).

**Table 2:** Disease-specific laboratory studies Ab: antibody, Ig: immunoglobulin AChR: acetylcholine receptor, MuSK: muscle-specific kinase, CASPR2-IgG: anticontactin-associated protein-like 2 immunoglobulin G, AGNA: anti-glial nuclear antibody, ANNA: anti-neuronal nuclear autoantibody, CRMP:  collapsin response mediator protein, DPPX: dipeptidyl-peptidase-like protein, IFA: indirect immunofluorescence assay, GABA-B-R, gamma-aminobutyric acid type B receptor, CBA: cell binding assay, GAD: glutamic acid decarboxylase, GFAP: glial fibrillary acidic protein, LGI: leucine-rich glioma-inactivated, NIF: neuronal intermediate filament, PCA: Purkinje cytoplasmic antibody, NDMA-R: N-methyl-D-aspartate receptor, mGluR1: metabotropic glutamate receptor 1, AMPAR: alpha-amino-3-hydroxy-5methyl-4-isoxazoleproprionic acid receptor

Study Name	Reference Value	Result
AChR-binding Ab	0.00-0.24 nmol/L	<0.03
AChR-blocking Ab	0-25%	24%
MuSK Antibody	<1.0 U/mL	<1.0
AChR-modulating Ab	0-45%	0%
CASPR2-IgG CBA	negative	negative
Amphiphysin Ab	<1:240	negative
AGNA-1	<1:240	negative
ANNA-1	<1:240	negative
ANNA-2	<1:240	negative
ANNA-3	<1:240	negative
CRMP-5-IgG	<1:240	negative
DPPX Ab IFA	negative	negative
GABA-B-R Ab CBA	negative	negative
GAD65 Ab Assay	< .02 nmol/L	0.01
GFAP IFA	negative	negative
IgLON5 IFA	negative	negative
LGI1-IgG CBA	negative	negative
mGluR1 Ab IFA	negative	negative
NIF IFA	negative	negative
NMDA-R Ab CBA	negative	negative
PCA-1	<1:240	negative
PCA-2	<1:240	negative
PCA-Tr	<1:240	negative
GQ1b Ab (IgG)	<1:100	<1:100
AMPAR Ab CBA	negative	negative

There remained a high index of suspicion for MG in view of an up to 12% incidence of seronegativity of the disease [2]. Ice pack testing was of low utility due to residual sensory deficits from prior craniotomy for tumor removal. Beta blocker was replaced with calcium channel blocker for rate control and he was started on pyridostigmine and steroids without improvement. In an effort to avoid an anaphylactic reaction, IVIG was not considered a viable treatment option given the patient's history of IgA deficiency. In addition, IgA-specific IVIG was unavailable at the treatment facility. Ultimately, it was decided the patient should undergo a total of ten plasma exchange sessions (PLEX) every other day followed by weekly sessions as an outpatient. The patient first exhibited mild improvement at session four and was discharged with physical therapy and instructions for weekly PLEX after session ten. At his neurology follow-up one week later he and his family reported only mildly improved symptoms. The patient continued weekly PLEX treatments for one month until he, unfortunately, passed away due to complications from acute hypoxic respiratory failure secondary to pneumonia and pulmonary edema.

## Discussion

Though MG can independently manifest as a paraneoplastic syndrome, the association with de novo diagnosis after starting pembrolizumab suggests a likely checkpoint-inhibitor-associated presentation of MG [3]. Our patient developed symptoms consistent with myasthenia gravis shortly after being treated with pembrolizumab, which included ptosis, dysarthria, cervical myalgia, generalized fatigue, and weakness. Immunochemical testing showed the patient to be seronegative for all disease-associated antibodies making pembrolizumab-induced myasthenia gravis a more likely diagnosis than primary myasthenia gravis. Our patient failed to show improvement with pyridostigmine and steroids. It was not until serial plasma exchanges were started that the patient experienced symptomatic relief, and even then, it was minimal. 

While effective, immunomodulator medications have their own set of adverse effects. Autoimmunity has been suggested to play a large role in which irAEs arise. As tumor cells are successfully destroyed, they release neoantigens that are recognized by antigen-presenting cells and activate the secondary immune system. Cross-reactivity between tumor neoantigens and host tissue has been proposed as a driving mechanism of irAE [4].

The most frequently noted immune-related adverse events involve inflammation of gastrointestinal, dermatologic, endocrine, or pulmonary organs [5]. Less commonly, neurological complications may arise. One study found an incidence of high-grade (Grade 3-4) irAE, including myasthenic syndromes, was less than 1% in patients receiving immune checkpoint inhibitors (ICI) [6]. Myasthenia gravis [7-11], encephalitis/meningitis [12], inflammatory polyradiculopathies [13] such as Guillain-Barre syndrome, and peripheral neuropathy have been described most commonly [14]. The significance of these associated neurological disorders resides in their high mortality rates. In a review of cases of irMG, a 29.8% mortality rate was observed with respiratory paralysis being the most common cause of death [15]. Although the pathology of why irAEs occur is generally well understood, it has been difficult to extrapolate the means by which a certain constellation of symptoms will be phenotypically expressed in a patient, and what risk factors predispose individuals to developing irAEs. Huang et al. also state that prior diagnosis of myasthenia gravis did increase the risk of the development of irMG. Prior MG diagnosis did not, however, increase mortality rates. It has been proposed that the difference in mortality between classical myasthenia gravis (~6%) and irMG is multifactorial, including the age of onset, concomitant malignancy, malignancy-related complications, and response to traditional immunotherapy [15].

Although these complications are rare, there is the possibility they become more prevalent as these therapies become common practice. With this in mind, it is evident that standardizing the guidelines for their treatment will become a growing need. As aforementioned, the Society for Immunotherapy of Cancer has given recommendations for the management of irMG. There have been attempts to retrospectively analyze the outcomes of cases treated using these guidelines, but the results varied widely [1,15]. The heterogeneity of these studies further highlights a lack of insight into how to manage these complications most effectively. 

Based on Common Terminology Criteria for Adverse Effects (CTCAE) version 5, our patient met the grade 3 criteria. Plasmapheresis or intravenous immunoglobulin may be considered for grade 3 and higher events according to the American Society of Clinical Oncology (ASCO) guidelines, although the National Comprehensive Cancer Network (NCCN) only recommends the addition of plasmapheresis or intravenous immunoglobulin if there is no improvement or with worsening symptoms after corticosteroids [16]. This was the case with our patient, but what was concerning was his lack of robust reversal of fatigue and ptosis over the course of his PLEX treatments. The patient’s minimal response to guideline-recommended therapy adds to the small sample size of patients that experience irMG and stresses the need for further studies to better standardize its treatment. His IgA deficiency also begs the question of what should be done for patients who have contraindications to therapies recommended by current guidelines.

## Conclusions

It is evident that although ICIs have provided effective treatment for devastating diseases, they also present the risk of untoward adverse effects. This case highlights the importance of maintaining a high level of suspicion for irMG and other irAE in patients on pembrolizumab and drugs in the same class. There is a clear need for further investigation and study into their propagation as it appears to be rather unpredictable and spontaneous at this time. By doing so, we will gain better insight into specific irAE and thereby be able to recognize those who are most susceptible to poor outcomes. As a result, there will be a future in which clinicians are more prepared to provide optimized therapy to patients treated with pembrolizumab or related medications.
